# Progression of deltamethrin resistance in *Rhipicephalus microplus* populations on communal farms of South Africa

**DOI:** 10.1007/s00436-025-08493-1

**Published:** 2025-05-02

**Authors:** William Diymba Dzemo, Songezo Mavundela

**Affiliations:** Department of Biological and Environmental Sciences, Faculty of Natural Sciences, Walter Sisulu University, Mthatha, 5117 South Africa

**Keywords:** Acaricide, Deltamethrin, Resistance establishment, Cattle ticks, *Rhipicephalus microplus*

## Abstract

This study was conducted to assess the progression of deltamethrin resistance in *Rhipicephalus (B.) microplus* populations over a 5-year period, from 2019 to 2023, on selected communal farms in the Eastern Cape Province, South Africa. Ticks were collected from three communal farms with a documented history of deltamethrin resistance in *R. (B.) microplus* populations in 2019. The sampled tick populations were tested for resistance using the adult immersion test (AIT). The data generated from the AIT indicated deltamethrin resistance, with oviposition inhibition percentages ≤ 95% at both the manufacturer’s recommended dose and twice the recommended dose. These findings confirm the establishment of deltamethrin resistance in *R. (B.) microplus* populations on the communal farms within the KSD local municipality, following over a decade of frequent and continuous use of this acaricide. Ticks on cattle, along with their control and acaricide resistance in tick populations, pose a significant threat to livestock production on communal farms in South Africa.

**Supplementary Information:**

The online version contains supplementary material available at 10.1007/s00436-025-08493-1.

## Introduction

Ixodid ticks and tick-borne diseases (TBDs) are a major challenge to livestock production in resource-limited communal farming areas of the Eastern Cape (EC) (Nyangiwe et al. 2021). On these farms, five tick species of significant veterinary importance have been sampled from cattle, namely *Amblyomma hebraeum*, *Rhipicephalus appendiculatus*, *Rhipicephalus evertsi evertsi*, *Rhipicephalus (Boophilus) decoloratus* and *Rhipicephalus (Boophilus) microplus*. The invasive *R.* (*B.*) *microplus* is reported to have displaced the indigenous *R.* (*B.*) *decoloratus* in most parts of the EC (Dzemo et al. 2023; Nyangiwe et al. 2013). The control and management of ticks and TBDs in communal areas of the EC of South Africa is done mainly through the regular dipping of animals at a frequency of two to four times in summer and none to two times per month in winter (Dzemo et al. 2024). The Eastern Cape provincial government, through the District Veterinary Services, provides acaricides to these dip tanks. Unfortunately, due to the procurement processes that rely on the bidding or tendering system, a selected chemical formulation is widely used over an extended period, which often leads to the selection and proliferation of resistant tick species (Dzemo et al. 2024). Consequently, farmers sometimes complement the government-provided dipping services with additional measures such as chemical acaricide sprays, chemicals poured-on the animals and injections (Dzemo et al. 2024).

Economic losses resulting from tick infestations and TBDs are estimated to exceed USD 33 million per year in South Africa (Makwarela et al. 2023). Globally, losses due to infestation with *R.* (*B.*) *microplus*, its associated diseases, and the cost of control are estimated at USD 13.9–18.7 billion per year (Hurtado & Giraldo-Ríos 2018). The ability of ticks to develop resistance rapidly to acaricides is one of the most significant factors contributing to their cost of control (Bourguet & Guillemaud 2016). Acaricide resistance is an evolutionary adaptation in tick populations, driven by the selection pressure of intensive and indiscriminate acaricide use. This practice results in a decreased susceptibility of the ticks to the acaricide in the field (Dzemo et al. 2022). A range of genetic, environmental and operational factors usually contribute to the development of resistance in tick populations in the field (Després et al. 2007). The majority of research on acaricide resistance in South Africa has tended to focus on case studies on the characterisation of resistance in field tick populations (Dzemo et al. 2023; Lovis et al. 2013; Ntondini et al. 2008; Yawa et al. 2022) and elucidation of mechanism of resistance to specific acaricide classes (Baron et al. 2015; Lovis et al. 2012; Robbertse et al. 2016). While these studies are crucial for monitoring and managing of tick-acaricide resistance (FAO 2004), they do not provide valuable insights on the pace of evolution for acaricide resistance (Gressel 2011). This study examined the progression of deltamethrin resistance in *R.* (*B.*) *microplus* populations over a quinquennium (between 2019 and 2023). In vitro bioassays were used to assess and compare the resistance status on selected communal farms of the King Sabata Dalindyebo local municipality (KSDLM), where deltamethrin resistance had been previously documented, as reported in Dzemo et al. (2023). This information is vital for the effective management of deltamethrin resistance in field tick populations and for ensuring that acaricide treatments remain effective over time.

## Material and methods

### Study area

**Fig. 1:**
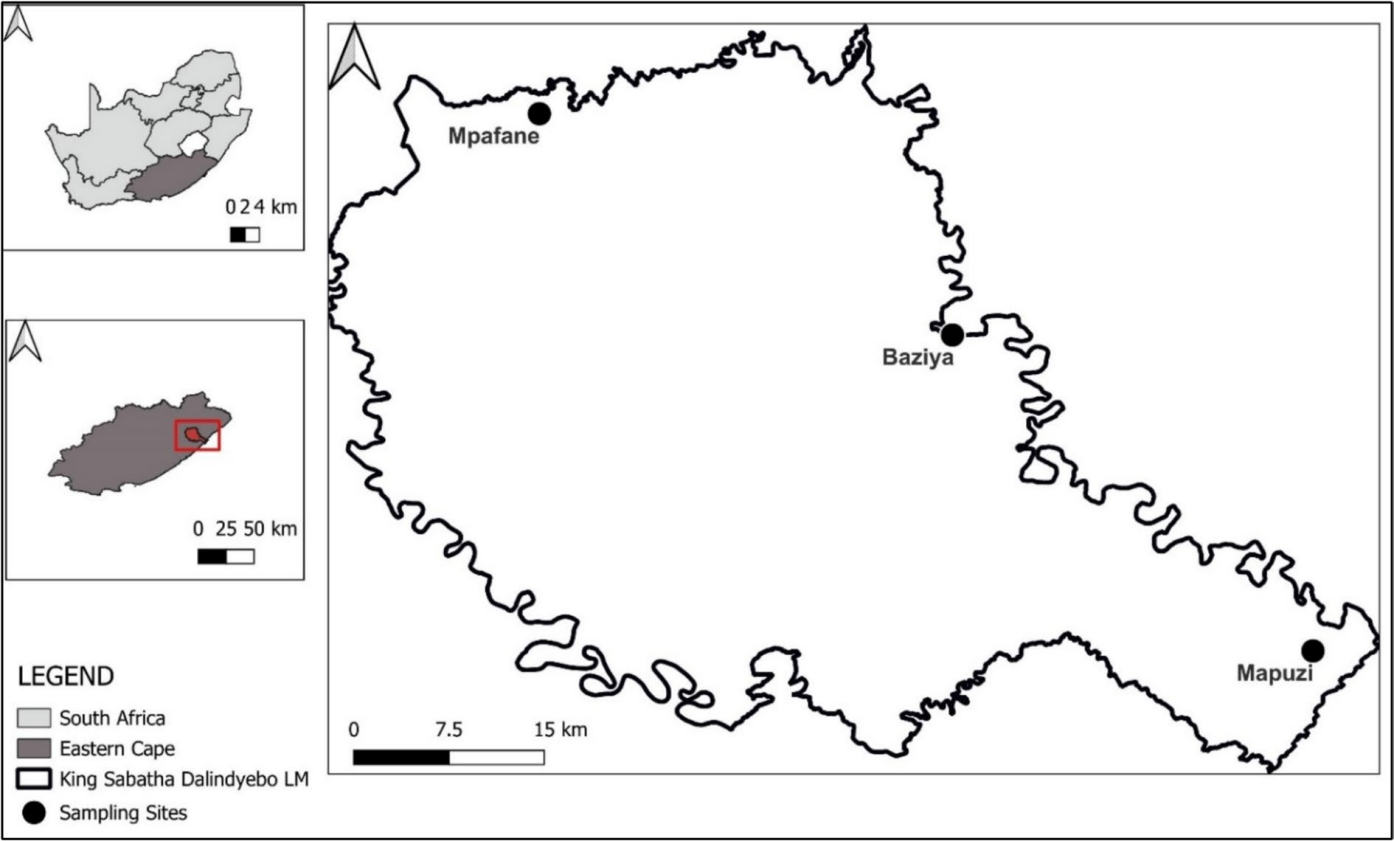
Communal farms and sampling sites from which ticks were collected

The study was conducted at three selected communal farming areas, namely Mapuzi, Baziya and Mpafane, which are in the KSDLM of the EC of South Africa (Fig. 1). The KSDLM has a landscape that consists of hills and mountains, with an average elevation of about 764 m. The climate changes with distance from the Indian Ocean, where the coastal areas have a tropical climate and the inland areas a temperate climate. The mean temperature ranges from 14.3 to 25.3 °C in summer months and 1.8 to 21.4 °C in winter months. The annual rainfall is approximately 871 mm in coastal areas and decreases as one moves inland (Dzemo et al. 2024). The vegetation of the KSDLM is made up of a mosaic of thick savannah bushes (in the coastal area) and grassland (in the inland area), with diverse grass species, including *Digitaria eriantha*, *Paspalum*, *Cynodon dactylon*, *Eragrostis plana* and *Festuca rubra* (Nyangiwe et al. 2013), that are suitable for livestock (sheep, goats, cattle) rearing. Most of the livestock production is under the informal subsistence farming system in which cattle are kept mostly in communal areas. In such areas, farmers have no exclusive land tenure, and natural resources such as grazing land are shared and managed collectively (Ibelli et al. 2012). Acaricide chemical formulations, including Deca-tix®3 (deltamethrin 2.5% m/v), Delete-X5® (deltamethrin 5% m/v) and Taktic® (amitraz 12.5% m/v), have been widely used at the communal dipping tanks to treat ticks on cattle. Other agricultural activities practiced within the municipality include crop farming and forestry (Dzemo et al. 2023).

## Tick collection, handling and identification

Tick collection was conducted between February and April 2023 at three dipping tanks on communal farms (Fig. 1), mirroring the tick collection period in 2019, by Dzemo et al. 2023. Prior to tick collection, verbal consent was obtained from community animal health workers and cattle owners and dates for tick collection were scheduled. On the day of tick collection, the dipping tank areas were visited early in the morning (05:00–07:30) before cattle were treated for ticks using a dip solution. Cattle from individual farmers (groups) were restrained in a cattle crush separately, and engorged adult female *Rhipicephalus* (*Boophilus*) spp. were collected from their preferred sites using steel fine forceps, from as many different cows as possible. Approximately 200 engorged adult female ticks (7–12 mm long) were sampled from each of the three communal farms, to allow for the number of dilutions and replications. In addition, a few male *R*. (*B*.) spp. were also collected for identification purposes.

The collected ticks were placed in perforated plastic containers for ventilation and then placed in a cooler box with ice packs to maintain a cold and humid environment, to reduce the chances of egg-laying during transportation. The ticks were then transported to the Zoology laboratory of the Walter Sisulu University. Upon arrival at the laboratory, the fully engorged adult female ticks were placed in a sieve (opening size 1000 µm) and washed with clean tap water to remove any eggs laid during transportation (Drummond et al. 1973; FAO 2004). Damaged or discoloured ticks, as well as those that had started laying eggs, were discarded. The remaining ticks were gently dried with an absorbent paper towel. Ticks collected from different farmers at each communal farm or dip-tank area were pooled and treated as a single collection from a farm.

The male and female ticks were examined under a dissecting microscope (STEMI DV4™, Oberkochen, Germany) and identified morphologically using the taxonomic keys described in Walker et al. (2003). Identification of *R*. (*B.*) *microplus* was confirmed based on distinguishing features, including the hypostomal teeth dentition and internal margin of palp article 1. In *R*. (*B.*) *microplus,* the hypostomal teeth dentition is arranged in 4 + 4 columns (Supplementary Fig. 1). Additionally, *R*. (*B.*) *microplus* lacks a protuberance bearing setae on the internal margin of palp article 1.

## Acaricide used in the study

Based on information from the local veterinary authorities, Deca-tix®3 (deltamethrin 2.5% m/v) and Delete®-X5 (deltamethrin 5% m/v) have been extensively used at the communal dipping tanks for over a period of 10 years. Delete®-X5, a synthetic pyrethroid, is currently being used for the treatment of ticks on cattle at the sampled communal farms. Delete®-X5 is available commercially in veterinary drug shops and has been verified for the concentration of its active ingredients by the South African National Pesticide Registration authority, under registration number G3279, Act 36 of 1947. The required quantity of Delete®-X5 for charging a plunge dip treatment of ticks on cattle is 500 ml per 1000 l of water. A 500 ml quantity of Delete®-X5 used for the control of ticks on cattle at the communal dipping tanks of the KSDLM was obtained from the local veterinary authorities for the in vitro assessment of tick-acaricide resistance. The acaricide was stored in the storeroom of the local veterinary clinic. Acaricide concentrations prepared from a stock solution (5000 ppm) for use in the study included 50 ppm (the manufacturer-recommended concentration considered the diagnostic dose, [DD]), 100 ppm (2DD), 25 ppm (0.5DD), and distilled water as the control. These acaricide concentrations were obtained by diluting the commercial formulations of Delete®-X5 in distilled water.

## Adult immersion test (AIT)

After morphological identification, 120 engorged female *R.* (*B.*) *microplus* of similar size (7–12 mm) were sorted into groups of ten and placed in 50-ml beakers. Each group was weighed using a digital laboratory scale (KERN ABJ-NM/ABS-N™, Balingen, Germany) and assigned to one of four treatments in triplicates. The masses obtained were analysed using one-way ANOVA at a 5% significance level to ensure no significant differences (*P* > 0.05) existed between the average mass of each treatment.

Each group of ten ticks was immersed and gently agitated in three different dilutions of deltamethrin (2DD, DD, 0.5DD), along with distilled water (control) in triplicates. The treatment duration was extended to 30 min, as recommended for optimal acaricide effectiveness (Klafke et al. 2012), similar to the modified AIT in FAO (2004). After treatment, ticks were dried and pasted ventrally onto double-sided tape inside petri dishes. Petri dishes were sealed and incubated under controlled conditions (27–28 °C, with 80–95% humidity) for 14 days to observe oviposition (Haque et al. 2014; Yessinou et al. 2018). Ticks that laid eggs were considered alive, while those that did not were deemed dead (FAO 2004). The following parameters were recorded and compared:

- Percentage of adult tick mortality per replicate
- The mass of eggs laid by treated ticks per replicate
- Index of fertility (IF)—a measure of the egg-laying capacity of the treated ticks expressed as the weight of eggs laid (mg)/weight of female ticks (mg)
- Percentage inhibition of oviposition (%IO) = [(IF control group − IF treated group)/(IF control group) × 100]

Acaricide concentrations were deemed effective when the efficacy (percentage of inhibition of oviposition) was higher than or equal to 95% (Drummond et al. 1973; FAO 2004).

## Data analysis

The AIT data was recorded in Microsoft® Excel® for Microsoft 365. The significance of the index of fecundity (IF) and percentage inhibition of oviposition (%IO) between groups was determined by one-way ANOVA at the 5% level of significance. Furthermore, a paired sample *t*-test was used to assess for differences in means of % inhibition of oviposition (IO) between the years 2019 and 2023.

## Results and discussion

The effect of immersing adult engorged females of *R.* (*B.*) *microplus* collected from the same communal farms in 2019 and 2023 into DD, 2DD and ½DD concentration of deltamethrin on reproductive parameters, including index of fertility (IF) and IO, is represented in Table 1. All three field tick populations collected from communal farms in 2023 showed resistance (% IO ≤ 95%) to deltamethrin at the DD and 2DD levels, signifying a reduced efficacy of the acaricide in preventing tick reproduction. Similarly, Dzemo et al. (2023) reported deltamethrin resistance in *R.* (*B.*) *microplus* populations on these same communal farms in Mapuzi, Mpafane and Baziya, as well as three additional farms in their 2019 study. It is worth noting that when comparing the IO values between these two studies, the level of resistance has relatively decreased. Field populations of *R.* (*B.*) *spp*. from communal farms in the EC have also been reported to exhibit resistance to cypermethrin (Mekonnen et al. 2002; Ntondini et al. 2008). More recently, cypermethrin-resistant field *Rhipicephalus (Boophilus)* spp. populations have been documented on communal farms in the Elundini, Senqu and Walter Sisulu local municipalities in the northeastern region of the Eastern Cape (Yawa et al. 2022).

**Table 1:** Comparison of the effect of deltamethrin on the index of fecundity (IF) and oviposition inhibition percentage (%IO) in *R.* (*B.*) *microplus* populations collected from communal farms in February to April 2019 and 2023

Index of fecundity (IF ± SE) Deltamethrin concentration (ppm)					
Communal farm	Year of data collection	100	50^a^	25	Control
Mpafane	2019	0.1130 ± 0.0114	0.1253 ± 0.0216	0.1483 ± 0.0158	0.3288 ± 0.0325
	2023	0.3967 ± 0.0850	0.3319 ± 0.1587	0.2044 ± 0.0368	0.4797 ± 0.0696
Baziya	2019	0.1804 ± 0.0382	0.2867 ± 0.0937	0.3058 ± 0.0100	0.3943 ± 0.0186
	2023	0.3533 ± 0.0587	0.4052 ± 0.0643	0.4222 ± 0.0273	0.5361 ± 0.0824
Mapuzi	2019	0.1150 ± 0.0206	0.1687 ± 0.0224	0.1733 ± 0.0127	0.2405 ± 0.0247
	2023	0.2008 ± 0.1020	0.3276 ± 0.0188	0.3564 ± 0.0164	0.4984 ± 0.0581
**Percentage inhibition of oviposition (%IO ± SE)**					
Mpafane	2019	63.51 ± 6.02	59.98 ± 9.61	54.59 ± 4.62	0.00 ± 0.00
	2023	8.75 ± 29.97	21.4060 ± 46.62	53.76 ± 14.38	0.00 ± 0.00
	*p*-value	*0.16*	*0.44*	*0.94*	-
Baziya	2019	55.00 ± 7.85	24.69 ± 7.83	22.35 ± 1.26	0.00 ± 0.00
	2023	27.79 ± 20.50	20.56 ± 19.20	17.25 ± 13.39	0.00 ± 0.00
	*p*-value	0.21	0.92	0.72	-
Mapuzi	2019	49.58 ± 10.58	28.07 ± 12.50	27.15 ± 5.72	0.00 ± 0.00
	2023	54.44 ± 25.11	32.80 ± 6.96	25.50 ± 12.79	0.00 ± 0.00
	*p*-value	*0.89*	*0.79*	*0.91*	-

^a^Manufacturer-recommended dose; *IF* index of fecundity, *%IO* oviposition inhibition percentage, *SE* standard error

Deltamethrin, along with other commercially available synthetic pyrethroid (SP) chemical compounds such as flumethrin and cypermethrin, had been used to supplement the state-funded dipping programme for more than 5 years (Dzemo et al. 2023). By the time the current study was conducted in 2023, deltamethrin and other SP chemical formulations had been in use for more than a decade on these farms. Synthetic pyrethroids are the most frequently used veterinary product in the EC of South Africa (Dzemo et al. 2023). The strong selective pressures from the prolonged and indiscriminate use of deltamethrin and other SP chemical formulations might have contributed to the predominance of tick populations that are no longer susceptible to deltamethrin or other SP compounds with a similar mode of action. Furthermore, Dzemo et al. (2024) identified several risk factors associated with tick-acaricide control failure on these communal farms, including the lack of acaricide rotation, irregular cattle treatment, high treatment frequency, weak strength of the dip solution and the poor structural state of the dip tanks.

A comparison of the mean % IO obtained at the DD and 2DD concentrations of deltamethrin between the 2 years shows no statistically significant difference (*P* > 0.05) (Table 1). This suggests that the level of deltamethrin resistance in *R.* (*B.*) *microplus* populations on the three farms had not changed significantly. It is worth noting that deltamethrin was applied on these farms once per month during winter and twice per month during summer. The treatment frequency exerted was sufficient selection pressure to drive the progression of acaricide resistance in *R.* (*B.*) *microplus* populations on these farms. Over the 5 years between assessments for deltamethrin resistance in these tick populations, *R.* (*B.*) *microplus* would have completed 15–30 reproductive cycles, depending on the environmental conditions (Sales et al. 2024). In highly resistant species, including *R.* (*B.*) *microplus*, the duration (mean years ± SD) between the introduction of deltamethrin and the first report of resistance is 15 ± 8.59 years (Brevik et al. 2018), which corresponds to 45–90 generations of ticks.

The rate of evolution of acaricide resistance is influenced by a number of factors, including inherent genetic variance due to mutations and initial gene frequencies of the resistance allele and its effectiveness (Brevik et al. 2018). Once these genetic differences arise, selection across generations might promote the accumulation and persistence of these variations (Lee & Gelembiuk 2008). Also, the number of reproductive cycles per year and the population size could influence the likelihood that populations develop resistance (Brevik et al. 2018). *Rhipicephalus* (*B.*) *microplus* is a one-host tick with a relatively rapid reproduction cycle, especially in regions with high levels of humidity and high temperatures, where its reproduction rate is the highest. Farms in humid areas would therefore have larger field tick populations and might have more chances for mutations leading to resistance. In addition, the possibility of ticks receiving sub-lethal doses can contribute to the progression of acaricide resistance, particularly when communal farmers attributed tick control failure to the weak strength of the dip solution (Dzemo et al. 2024). At very low acaricide application rates, a certain subpopulation of ticks may receive sub-lethal doses and survive. These survivors are more likely to exhibit higher mutation rates, potentially accelerating the development of acaricide resistance. Furthermore, the lack of a significant change in deltamethrin resistance in *R.* (*B.*) *microplus* populations may suggest that deltamethrin resistance has become endemic on these farms.

## Conclusion

Findings from this study confirm the continued presence of deltamethrin resistance in *R*. (*B.*) *microplus* populations on the communal farms of the KSD local municipality, following more than a decade of its frequent and continuous use. Therefore, it is crucial to reassess its usage on these farms. The implementation of an alternative acaricide formulation from different chemical classes should be strongly considered to enhance tick management strategies.

## Supplementary Information

Below is the link to the electronic supplementary material.

ESM 1 (DOCX 118 KB)

## Data Availability

No datasets were generated or analysed during the current study.
