# Supplementary material for: Progression of deltamethrin resistance in Rhipicephalus microplus populations on communal farms of South Africa
Source: Parasitol Res. 2025 May 2;124(5):48. doi: 10.1007/s00436-025-08493-1 (PMC12048425; doi:10.1007/s00436-025-08493-1)
Supplement: Supplementary file 1 — (DOCX 118 KB) [file 436_2025_8493_MOESM1_ESM.docx]

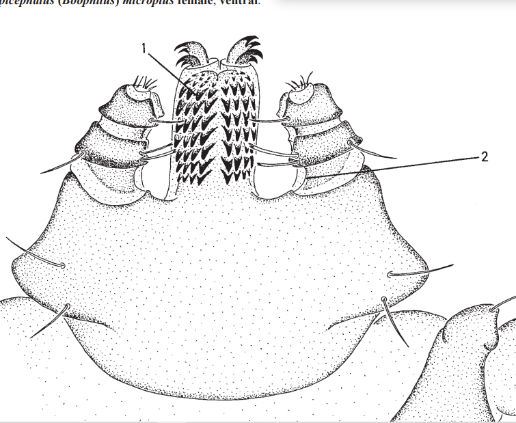


Supplementary Figure 2. Morphological keys used in the identification of *R.* (*B.*) *microplus*, Walker et al. (2003): 4+4 hypostomal teeth dentition (1) and palp article 1 (2).
